# Lovastatin‐mediated MCF‐7 cancer cell death involves LKB1‐AMPK‐p38MAPK‐p53‐survivin signalling cascade

**DOI:** 10.1111/jcmm.14879

**Published:** 2019-12-10

**Authors:** Shiu‐Wen Huang, I‐Tsu Chyuan, Ching Shiue, Meng‐Chieh Yu, Ya‐Fen Hsu, Ming‐Jen Hsu

**Affiliations:** ^1^ Department of Medical Research Taipei Medical University Hospital Taipei Taiwan; ^2^ Department of Pharmacology School of Medicine College of Medicine Taipei Medical University Taipei Taiwan; ^3^ Graduate Institute of Medical Sciences College of Medicine Taipei Medical University Taipei Taiwan; ^4^ Department of Internal Medicine Cathay General Hospital Taipei Taiwan; ^5^ Department of Medical Research Cathay General Hospital Taipei Taiwan; ^6^ School of Medicine, College of Medicine Fu Jen Catholic University New Taipei Taiwan; ^7^ Division of General Surgery Department of Surgery Landseed Hospital Taoyuan Taiwan; ^8^ Cell Physiology and Molecular Image Research Center Wan Fang Hospital Taipei Medical University Taipei Taiwan

**Keywords:** breast cancer, liver kinase B1, lovastatin, p53, surviving

## Abstract

There is increasing evidence that statins, which are widely used in lowering serum cholesterol and the incidence of cardiovascular diseases, also exhibits anti‐tumour properties. The underlying mechanisms by which statins‐induced cancer cell death, however, remain incompletely understood. In this study, we explored the anti‐tumour mechanisms of a lipophilic statin, lovastatin, in MCF‐7 breast cancer cells. Lovastatin inhibited cell proliferation and induced cell apoptosis. Lovastatin caused p21 elevation while reduced cyclin D1 and survivin levels. Lovastatin also increased p53 phosphorylation, acetylation and its reporter activities. Results from chromatin immunoprecipitation analysis showed that p53 binding to the survivin promoter region was increased, while Sp1 binding to the region was decreased, in MCF‐7 cells after lovastatin exposure. These actions were associated with liver kinase B1 (LKB1), AMP‐activated protein kinase (AMPK) and p38 mitogen‐activated protein kinase (p38MAPK) activation. Lovastatin's enhancing effects on p53 activation, p21 elevation and survivin reduction were significantly reduced in the presence of p38MAPK signalling inhibitor. Furthermore, LKB1‐AMPK signalling blockade abrogated lovastatin‐induced p38MAPK and p53 phosphorylation. Together these results suggest that lovastatin may activate LKB1‐AMPK‐p38MAPK‐p53‐survivin cascade to cause MCF‐7 cell death. The present study establishes, at least in part, the signalling cascade by which lovastatin induces breast cancer cell death.

## 1 INTRODUCTION

Breast cancer is the most frequently diagnosed malignancy and the leading cause of cancer‐related death among females worldwide.^1^ Treatment of breast cancer is typically determined by staging information and histological subtype. The current therapeutic strategy for breast cancer is multidisciplinary. Patients may receive a combination of the modalities including surgical resection, radiotherapy, chemotherapy, hormonal therapy, target therapy and immunotherapy. However, the treatment protocols are sometimes too complicated or even toxic for the patients to eradicate breast cancer. Treatment non‐compliance or premature discontinuation of therapy also negatively affects outcomes.^2^, ^3^ As the high mortality rate of aggressive breast cancers, developing novel agents or strategies is an urgent need to improve therapeutic outcomes.

Beyond suppressing apoptosis, the smallest member of inhibitor of apoptosis protein (IAP) family, survivin, also regulates a variety of cellular processes such as mitosis, migration, angiogenesis and chemo‐resistance.^4^, ^5^, ^6^ Survivin is largely undetectable in most normal, terminally differentiated tissues with notable exceptions of vascular endothelial or haematopoietic cells. In contrast, it is highly expressed in most human cancers 7, 8 and its expression is associated with tumour progression and poor clinical outcome.^8^, ^9^, ^10^ Moriai et al^11^ recently showed that targeting survivin may overcome tamoxifen resistance in breast cancer cells. It indicates that survivin represents a promising therapeutic target for cancer treatment.^8^

Survivin expression is mainly regulated at the level of transcription. Many transcription factors such as specificity protein 1 (Sp1),^12^ signal transducer and activator of transcription 3 (STAT3)^13^ and hypoxia‐inducible factor‐1α (HIF‐1α) contribute to survivin induction. In contrast, tumour suppressor p53 may counteract Sp1 binding to the promoter region and thereby reduce survivin expression.^14^, ^15^ As p53 is an essential and negative regulator of survivin, pharmacological approaches targeting p53‐survivin signalling might be a promising therapeutic strategy for intervention of cancer.

Statins, the 3‐hydroxy‐3‐methylglutaryl‐coenzyme A (HMG‐CoA) reductase inhibitors, are widely used in treating hyperlipidemia and lowering risk of cardiovascular disease or its related complications.^16^, ^17^ Beyond lipid‐lowering activity, accumulating evidence demonstrates that statins also exhibit anti‐inflammatory and anti‐tumour activities.^18^, ^19^ Statins caused cell cycle arrest or apoptosis in various cancer types such as colorectal cancer, head and neck squamous cell carcinoma (HNSCC), lung cancer and breast cancer.^14^, ^20^, ^21^, ^22^ In experimental animal models, statins are capable of suppressing angiogenesis, tumour invasion and metastasis.^23^, ^24^ Epidemiologic studies also showed the concomitant use of statins might be beneficial to cancer outcomes.^25^ Although the pre‐clinical and clinical data remain controversial, statins still attract considerable attention for its therapeutic potential in the treatment of cancer.^26^, ^27^, ^28^, ^29^ Inhibition of HMG‐CoA reductase, which leads to protein geranylgeranylation, contributes to statins‐induced tumour cell death.^30^ However, statins also exhibit anti‐tumour effects through multiple mechanisms of action independent of the cholesterol‐lowering property. Statins are capable of causing G0/G1 or G2/M arrest or activating intrinsic apoptotic pathway via regulating Bcl‐2 protein family.^20^, ^31^ Statins may also target histone deacetylases (HDACs) to alter protein acetylation status to reduce cell proliferation and in vivo tumour growth.^20^, ^32^ Characterizing statins’ anti‐tumour mechanisms will aid in their proper application as anti‐cancer therapeutics in the future. Recent studies demonstrated that stains usage reduces breast cancer‐specific mortality in patients with breast cancer.^33^, ^34^ Kotamraju et al^35^ reported that statins activate arginase‐dependent pathways to cause cell death in breast cancer cells. Induction of oxidative stress,^36^ stimulation of inducible nitric oxide synthase (iNOS)^35^ or alterations of Bax and Bcl‐2 levels^37^ has been explored as a possible cause of statins’ cytotoxicity in MCF‐7 breast cancer cells. Modulation of cellular hyperpolarization and gap junctional intercellular communication (GKIC) function may also pay a role in anti‐proliferative and apoptotic effects of lovastatin in MCF‐7 cells.^38^ Moreover, several signalling cascades including p21 induction^14^, ^20^ and survivin reduction^11^ seem to be involved in lovastatin‐induced MCF‐7 cell death. However, the precise mechanisms involved in statins’ actions against breast cancer remain incompletely understood. In this study, we aim to explore the mechanisms underlying lovastatin‐induced survivin reduction and cell death in MCF‐7 breast cancer cells.

## 2 MATERIALS AND METHODS

### 2.1 Reagents

Transfection reagent, Turbofect™, MEM, DMEM, DMEM/F12 or RPMI medium, foetal bovine serum (FBS), TrypLE™ and all cell culture reagents were purchased from Invitrogen. 3‐[4,5‐dimethylthiazol‐2‐yl]‐2, 5‐diphenyltetrazolium bromide (MTT) and dimethyl sulfoxide (DMSO) were from Sigma‐Aldrich. Lovastatin, simvastatin, pravastatin and p38 inhibitor III (a p38 inhibitor) were purchased from Calbiochem. The vehicle used as controls for the drug experiments in this study is DMSO. Antibodies against normal IgG (Santa Cruz Cat# SC‐2025), LKB1 (Santa Cruz Cat# SC‐32245) and p21 (Santa Cruz Cat# SC‐756) were from Santa Cruz Biotechnology. Antibodies against p53 (Cell Signaling Technology Cat# 2527), p53 phosphorylated at Ser15 (Cell Signaling Technology Cat# 9284), p53 acetylated at Lys379 (K379) (Cell Signaling Technology Cat# 2570), Sp1 (Cell Signaling Technology Cat# 9389), LKB1 phosphorylated at Ser428 (Cell Signaling Technology Cat# 3482), p38MAPK (Cell Signaling Technology Cat# 9217), p38MAPK phosphorylated at Thr180/Tyr182 (Cell Signaling Technology Cat# 9211), AMPKα phosphorylated at Thr172 (Cell Signaling Technology Cat# 2535) and survivin (Cell Signaling Technology Cat# 2808) were from Cell Signaling. Antimouse and anti‐rabbit IgG conjugated horseradish peroxidase antibodies, as well as antibodies against AMPKα (GeneTex Cat# GTX113251), cyclin D1 (GeneTex Cat# GTX108624), α‐tubulin (GeneTex Cat# GTX628802), GAPDH (GeneTex Cat# GTX100118) and Myc tag (GeneTex Cat# GTX29106) were from GeneTex Inc The enhanced chemiluminescence detection kit was from Millipore. All materials for immunoblotting were purchased from Bio‐Rad. Dr Morris Birnbaum (HHMI) kindly provided the construct AMPK dominant negative mutant (AMPK‐DN). Construct of PG13‐luc with p53 binding sites (p53‐luc, Addgene plasmid #16642) and p21/WAF1 promoter luciferase construct (p21 pro‐luc, Addgene plasmid # 16451) as described previously^39^ were kindly provided by Dr Bert Vogelstein. Renilla‐luc and the Dual‐Glo luciferase assay system were purchased from Promega. All other chemicals were obtained from Sigma‐Aldrich.

### 2.2 Cell culture

MCF‐7, MDA‐MB‐231 and T47D cell lines were obtained from the Bioresource Collection and Research Center (Hsinchu, Taiwan). MDA‐MB‐468 cell line was kindly provided by Prof. Wei‐Chien Huang (Graduate Institute of Biomedical Sciences, China Medical University, Taichung, Taiwan). MCF‐7 cells were maintained in MEM medium containing 10% FCS, 100 μg/mL streptomycin and 100 U/mL of penicillin G in a humidified 37°C incubator. Other cells were maintained in DMEM (MDA‐MB‐231), RPMI1640 (T47D) or DMEM/F12 (MDA‐MB‐468) medium containing 10% FCS, 100 μg/mL streptomycin and 100 U/mL of penicillin G in a humidified 37°C incubator. A PCR Mycoplasma Detection Kit (Abm) was used to confirm that MCF‐7, MDA‐MB‐468 and MDA‐MB‐231 are negative for mycoplasma contamination. We have also performed the STR profiling to confirm identity of MCF‐7 and MDA‐MB‐231 cell lines (Supporting Information).

### 2.3 Cell viability (MTT) assay

The colorimetric 3‐(4,5‐dimethylthiazol‐2‐yl)‐2,5‐diphenyl tetrazolium bromide (MTT) assay was employed to determine cell viability as described previously.^40^

### 2.4 Flow cytometry

Cells were treated with lovastatin at indicated concentrations for 24 or 48 hours (h). Cells were washed twice with PBS and fixed in 70% ethanol at 0°C for another 24 hours. After washing with phosphate‐citric acid buffer, cells were stained by staining buffer (25 μg/mL PI, 100 μg/mL RNase A and 0.1% Triton X‐100) in the dark for 30 min. Flow cytometry was performed using the FACScan and Cellquest program (BD Biosciences). The percentage of PI‐stained cells in the subG1 (Apoptosis, Apo), G0/G1, S or G2/M region was analysed using the ModFit (BD Biosciences) or FCS Express (De Novo Software) program.

### 2.5 Immunoblotting

Cells were harvested in lysis buffer (10 mmol/L Tris [pH 7.0], 140 mmol/L NaCl, 0.5% NP‐40, 0.2 mmol/L leupeptin, 0.05 mmol/L pepstatin A and 2 mmol/L PMSF). Equal amounts of protein samples were subjected to SDS‐PAGE and transferred onto a nitrocellulose membrane (Pall Corporation). After blocking in a 5% non‐fat milk‐containing blocking buffer for 1 hour, proteins were recognized using specific primary antibodies followed by horseradish peroxidase‐conjugated secondary antibodies. The enhanced chemiluminescence was employed to detect immunoreactivity according to manufacturer's instructions. A computing densitometer with a scientific imaging system (Biospectrum AC System, UVP) was used to obtain the quantitative data.

### 2.6 Reverse‐transcription polymerase chain reaction (RT‐PCR)

MCF‐7 cells with or without treatments were harvested and total RNA was isolated for complementary DNA (cDNA) synthesis as described previously.^13^ The RT‐PCR was then conducted following the manufacturer's instructions (Super Script On‐Step RT‐PCR system, Invitrogen). Primers used for amplification of the survivin and GAPDH fragments were as follows: survivin sense, 5′‐gcc ttt cct taa agg cca tc‐3′; survivin anti‐sense, 5′‐aac cct tcc cag act cca ct‐3′; GAPDH sense, 5′‐gtc agt ggt gg acct gac ct‐3′; and GAPDH anti‐sense, 5′‐agg ggt cta cat ggc aac tg‐3′. GAPDH was used as the internal control. The PCR was performed with the following conditions: 30 cycles of a 30‐seconds denaturation step at 94°C, a 30‐seconds annealing step at 56°C, and a 45‐seconds extension step at 72°C to amplify survivin and GAPDH cDNA. The amplified fragment sizes for survivin and GAPDH were 187 and 420 bp, respectively. PCR products were run on an agarose gel, stained with ethidium bromide and visualized by ultraviolet illumination.

### 2.7 Chromatin immunoprecipitation (ChIP) analysis

After different treatments, MCF‐7 cells were cross‐linked with 1% formaldehyde at 37°C for 10 minutes. Cells were rinsed with ice‐cold PBS and harvested in SDS lysis buffer followed by sonication five times for 15 seconds each. Cells were centrifuged for 10 minutes, and supernatants were collected and diluted in ChIP dilution buffer. An aliquot of each sample was used as ‘Input’ in the PCR analysis. The remainder of the soluble chromatin was immunoprecipitated with normal IgG, p53 or Sp1 antibodies (Santa Cruz Biotechnology) at 4°C for 18 hours. Protein A‐Magnetic Beads (Millipore) were added and incubated for another 2 hours at 4°C with a gentle rotation to collect the immune complexes. The complexes were sequentially washed in the following washing buffers: low salt immune complex washing buffer, high‐salt immune complex washing buffer and LiCl immune complex washing buffer. After wash with Tris‐EDTA buffer two times, the complexes were eluted twice for 100 µL aliquots of elution buffer each. The cross‐linked chromatin complex was reversed by incubation with 0.2 mol/L NaCl and heating at 65°C for 4 hours. DNA was purified using GP^TM^ DNA purification spin columns (Viogene). PCR was performed using PCR Master Mix (Promega), according to the manufacturer's protocol. Ten per cent of the total purified DNA was used for the PCR in a 50‐µL reaction mixture. The 228‐bp *survivin* promoter fragment between −264 and −37 was amplified using PCR with the following primer pairs: sense: 5′‐ttc ttt gaa agc agt cga gg‐3′ and anti‐sense: 5′‐tca aat ctg gcg gtt aat gg‐3′. This was done with an initial denaturation at 95°C for 5 minutes, 30‐cycles of 30 seconds at 95°C, 30 seconds at 56°C and 45 seconds at 72°C and final extension for another 10 minutes at 72°C. The PCR products were analysed by 1.5% agarose gel electrophoresis.

### 2.8 Transfection in MCF‐7 cells

MCF‐7 cells (7 × 10^4^ cells/well) were transfected with p21 pro‐luc or p53‐luc plus renilla‐luc for reporter assay or transfected with pcDNA, AMPK‐DN, negative control siRNA or LKB1 siRNA for immunoblotting performed with Turbofect transfection reagent (Invitrogen) according to manufacturer's instructions.

### 2.9 Reporter assay (Dual‐Glo luciferase assay)

After transfection with reporter constructs plus renilla‐luc, MCF‐7 cells with or without treatments were harvested. The luciferase reporter activity was determined using a Dual‐Glo luciferase assay system kit (Promega) according to manufacturer's instructions and was normalized based on renilla luciferase activity.

### 2.10 Suppression of LKB1 expression

Target gene suppression was performed as previously described.^13^ For *LKB1* suppression, pre‐designed siRNA targeting the human *LKB1* or negative control siRNA was purchased from Sigma‐Aldrich (St Louis, MO, USA). The siRNA oligonucleotides were as follows: negative control siRNA, 5′‐gaucauacgugcgaucaga‐3′ and *LKB1* siRNA, 5′‐aaucagcugacagaaguac‐3′.

### 2.11 Randomization and blinding

The same cell (MCF‐7 cell) was used to evaluate the effects of lovastatin versus the related control in every single experiment. Therefore, formal randomization was not employed. In addition, we have different people conducting experiments (operator) and analysing data (analyst) for blinding.

### 2.12 Data and statistical analysis

In the present study, the data and statistical analysis comply with the recommendations on experimental design and analysis in pharmacology.^41^ Results are expressed as mean ± standard error of mean (SEM) (n ≥ 5), where 'n' refers to independent values, and not replicates. Normalization was performed to compare the differences after the treatment to control for unwanted sources of variation and to reveal relevant trends. Statistical analysis was performed using SigmaPlot 10 (Build 10.0.0.54; Systat Software). Statistical comparisons between two groups were evaluated by unpaired Student's t test for parametric analysis or Mann‐Whitney test for non‐parametric analysis. Statistical comparisons among more than two groups were evaluated by one‐way analysis of variance (ANOVA) with Tukey's post hoc test for parametric analysis or Kruskal‐Wallis test followed by Dunn's multiple comparison for non‐parametric analysis. Post hoc tests were run only if F achieved *P* < .05, and there was no significant inhomogeneity. A *P* value smaller than .05 was defined as statistically significant.

## 3 RESULTS

### 3.1 Lovastatin inhibited cell proliferation and induced apoptosis in MCF‐7 cells

**Figure 1:**
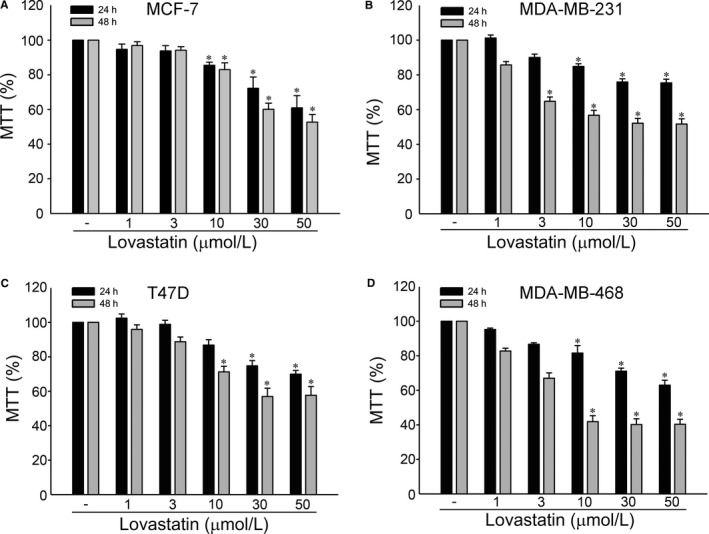
Lovastatin‐reduced cell viability in breast cancer cells. MCF‐7 (A), T47D (B), MDA‐MB‐231 (C) or MDA‐MB‐468 (D) breast cancer cells were treated with vehicle or lovastatin at indicated concentrations for 24 or 48 h. Cell viability was determined by an MTT assay. Each column represents the mean ± SEM of six independent experiments performed in duplicate (Statistically significant differences were determined using the Kruskal‐Wallis test. *P < .05, compared with the control group). Technical replicates were used to ensure the reliability of singe values for each experiment

**Figure 2:**
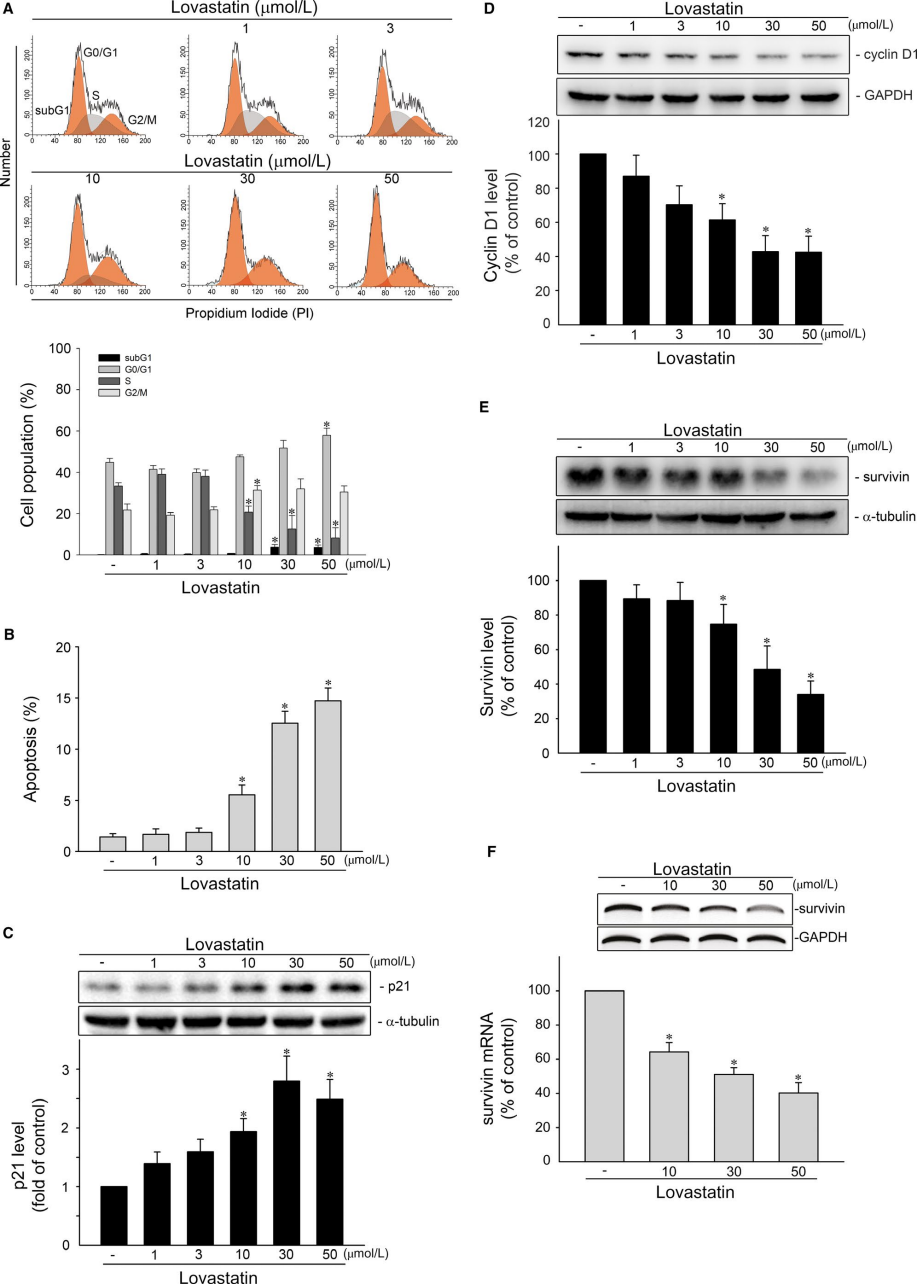
Lovastatin caused survivin reduction and apoptosis in MCF‐7 cells. A, Cells were treated with vehicle or lovastatin at indicated concentrations for 24 h. The percentage of propidium iodide‐stained cells in subG1, G0/G1, S and G2/M phases was analysed by flow cytometry as described in the ‘Materials and Methods’ section. Each column represents the mean ± SEM of four independent experiments (Statistically significant differences were determined using one‐way ANOVA, with Tukey's post hoc test. *P < .05, compared with the control group). B, Cells were treated as described in (A) for 48 h. The percentage of propidium iodide‐stained cells in apoptosis (subG1) region was analysed by flow cytometry as described in the ‘Materials and Methods’ section. Each column represents the mean ± SEM of four independent experiments (Statistically significant differences were determined using one‐way ANOVA, with Tukey's post hoc test. *P < .05, compared with the control group). MCF‐7 cells were treated with vehicle or lovastatin at indicated concentrations for 24 h. Protein levels of p21 (MW 21 kD) (C), cycin D1 (MW 36 kD) (D), survivin (MW 16 kD) (E), α‐tubulin (MW 52 kD) and GAPDH (MW 37 kD) were determined by immunoblotting. Each column represents the mean ± SEM of eight independent experiments (Statistically significant differences were determined using the Kruskal‐Wallis test. *P < .05, compared with the control group). F, Cells were treated with vehicle or lovastatin at indicated concentrations for 6 h. The survivin mRNA level was determined by an RT‐PCR as described in the ‘Materials and Methods’ section. Each column represents the mean ± SEM of three independent experiments (Statistically significant differences were determined using the Mann‐Whitney test. *P < .05, compared with the control group)

Similar to our previous studies, we usually select several cancer cell lines with different tumour subtypes or genetic background to confirm the cellular setting for our study. In this study, we selected MCF‐7, T47D, MDA‐MB‐231 and MDA‐MB‐468 cells. MCF‐7 and T47D are luminal subtype breast cancer cell lines while MDA‐MB‐231 and MDA‐MB‐468 are triple‐negative breast cancer cell lines. Among these four cell lines, mutant p53‐harbouring MDA‐MB‐231 and MDA‐MB‐468 cells exhibit high basal levels of STAT3 Y705 phosphorylation. In contrast, the basal STAT3 Y705 phosphorylation level is low in MCF‐7 cells, which retain functional p53. STAT3 is capable of up‐regulating survivin expression while p53 plays a negative regulatory role in survivin expression. We used MTT assay to examine the effects of lovastatin, a lipophilic statin, on cell viability in these four cell lines. As shown in Figure 1, lovastatin is capable of reducing cell viability in MCF‐7 (Figure 1A), T47D (Figure 1B), MDA‐MB‐231 (Figure 1C) and MDA‐MB‐468 (Figure 1D) cells in concentration‐ and time‐dependent manners. It raises the possibility that lovastatin may alter p53 or STAT3 signalling resulting in survivin reduction and cell death in breast cancer cells. In this study, we aimed to establish the p53‐related mechanisms underlying lovastatin‐induced survivin reduction and cell death in breast cancer cells. Therefore, we selected MCF‐7 cells to explore lovastatin's actions in inducing breast cancer cell death in the following experiment. Flow‐cytometric analysis with propidium iodide (PI) labelling was used to determine whether lovastatin affects cell cycle progression or induces apoptosis in MCF‐7 cells. As shown in Figure 2A, the percentage of PI‐stained cells in the S region was significantly reduced after 24 hours treatment of lovastatin at concentrations ranging from 10 to 50 μmol/L. These effects were accompanied by a concomitant increased in the percentage of PI‐stained cells in the G0/G1 region (Figure 2A). Lovastatin at 10 or 30 μmol/L slightly increased the percentage of PI‐stained cells in the G0/G1 region while lovastatin at concentration of 50 μmol/L significantly caused G0/G1 cell cycle arrest (Figure 2A). Longer exposure to lovastatin (48 hours) further increased the percentage of PI‐stained cells in the sub‐G1 (apoptosis) region (Figure 2B). Similar results were observed in MCF‐7 cells exposed to another lipophilic statin, simvastatin. In contrast, pravastatin, a hydrophilic statin, was without effects (Figure S1). These results indicate that lovastatin and simvastatin had similar potency in inhibiting cell proliferation and inducing apoptosis in MCF‐7 cells. In the following experiments, we selected lovastatin to explore its underlying mechanisms in causing MCF‐7 cell death.

### 3.2 Lovastatin caused p21 elevation and survivin reduction in MCF‐7 cells

It is believed that cyclin‐dependent kinase (CDK) inhibitor p21, cyclin D1, and survivin, a member of IAP family, play essential roles in regulating cell cycle progression. In addition, survivin reduction causes cell cycle arrest and death in a variety of cancer cells.^13^, ^14^, ^20^, ^42^ We thus determined whether lovastatin modulates the expression of these proteins in MCF‐7 cells. As shown in Figure 2C, treatment of cells with lovastatin significantly caused p21 induction. This effect was accompanied by decreased cyclin D1 levels (Figure 2D). Similar to previous reports,^11^, ^37^ we noted that exposure to lovastatin also led to a significant reduction in survivin protein (Figure 2E) and mRNA (Figure 2F) levels. It appears that lovastatin may negatively regulate survivin expression at the transcriptional level. In addition, Moriai et al^11^ demonstrated that knockdown survivin by survivin siRNA causes MCF‐7 cell apoptosis. These findings suggest that lovastatin‐induced MCF‐7 cell death may involve p21 elevation and survivin reduction. Moreover, lovastatin also reduced survivin levels in MDA‐MB‐231 and MDA‐MB‐468 triple‐negative breast cancer (TNBC) cells (Figure S2).

### 3.3 Lovastatin‐induced p53 activation in MCF‐7 cells

**Figure 3:**
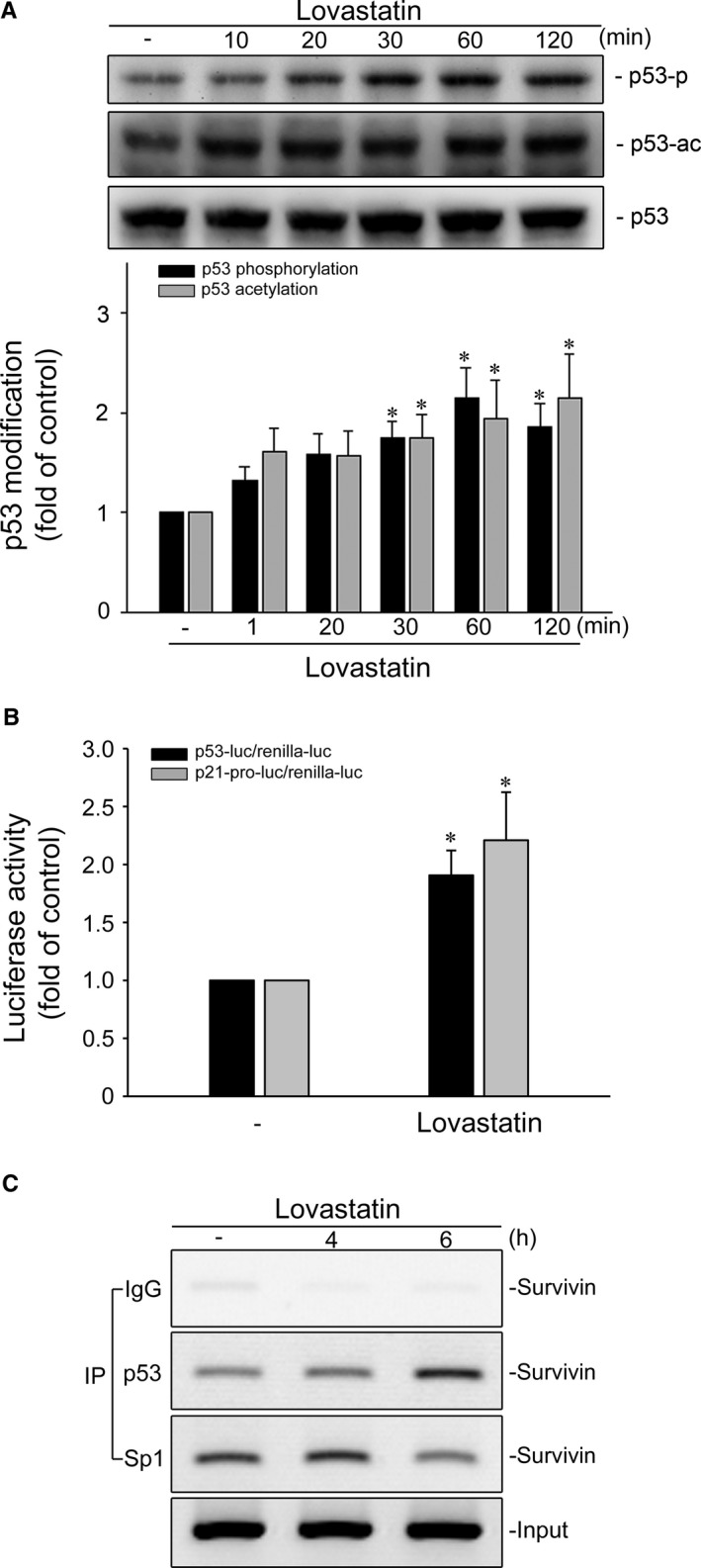
Lovastatin caused p53 activation in MCF‐7 cells. A, MCF‐7 cells were treated with vehicle or lovastatin at 30 μmol/L for indicated periods. The phosphorylation or acetylation status of p53 (MW 53 kD) was determined by immunoblotting. Each column represents the mean ± SEM of seven independent experiments (Statistically significant differences were determined using the Kruskal‐Wallis test. *P < .05, compared with the control group). B, Cells were transiently transfected with PG13‐luc (p53‐luc) or p21 promoter reporter construct (p21‐pro‐luc) plus renilla‐luc for 24 h followed by the treatment with lovastatin at 30 μmol/L for another 24 h. Reporter assay was performed as described in the ‘Materials and Methods’ section. Each column represents the mean ± SEM of five independent experiments performed in duplicate (Statistically significant differences were determined using the Mann‐Whitney test. *P < .05, compared with the control group). (C) Cells were treated with vehicle or lovastatin at 30 μmol/L for the indicated periods. A ChIP assay was performed as described in the ‘Materials and Methods’ section. Typical traces representative of five independent experiments with similar results are shown

Transcription factor p53 is deleted or mutated in approximately half of all human cancers, demonstrating the crucial role of p53 in tumour suppression.^43^, ^44^, ^45^ p53 suppresses tumour growth through regulating various target genes with diverse biological functions including cell cycle arrest and apoptosis.^39^, ^46^ p53 could up‐regulate p21 expression via activating p21 promoter.^39^ In contrast, p53 may counteract Sp1 binding to the promoter region (−264 to −37) and, thereby, repress survivin expression.^15^, ^20^, ^47^ Therefore, we examined the effects of lovastatin on p53 Ser15 phosphorylation and Lys379 acetylation, which are key steps in p53 activation.^48^, ^49^ As shown in Figure 3A, lovastatin exposure was associated with increases in p53 Ser15 phosphorylation and Lys379 acetylation in a time‐dependent manner. We also determined whether lovastatin increases p53 transcriptional activities performed with a reporter construct containing a p53 DNA‐binding site upstream of a basal promoter linked to a luciferase reporter gene (PG13‐luc/p53‐luc).^39^ As shown in Figure 3B, cells treated with lovastatin for 24 hours had a significant increase in p53‐luciferase activity. Lovastatin also caused an increase in p21 promoter luciferase activity (Figure 3B). We next performed the ChIP experiment to examine whether p53 or Sp1 binding to the endogenous *survivin* promoter region is altered in response to lovastatin. As shown in Figure 3C, lovastatin increased p53 binding to the *survivin* promoter region, and this was accompanied by a decrease in Sp1 binding to the promoter region. These results suggest that lovastatin may activate p53, leading to survivin reduction in MCF‐7 cells. In addition to MCF‐7 cells, which retains functional p53, lovastatin is capable of inactivating STAT3 (Figure S3), which leads to survivin reduction, in mutant p53‐harbouring MDA‐MB‐231 and MDA‐MB‐468 TNBC cells.

### 3.4 p38MAPK mediates lovastatin‐induced p53 phosphorylation, p21 elevation and survivin reduction in MCF‐7 cells

**Figure 4:**
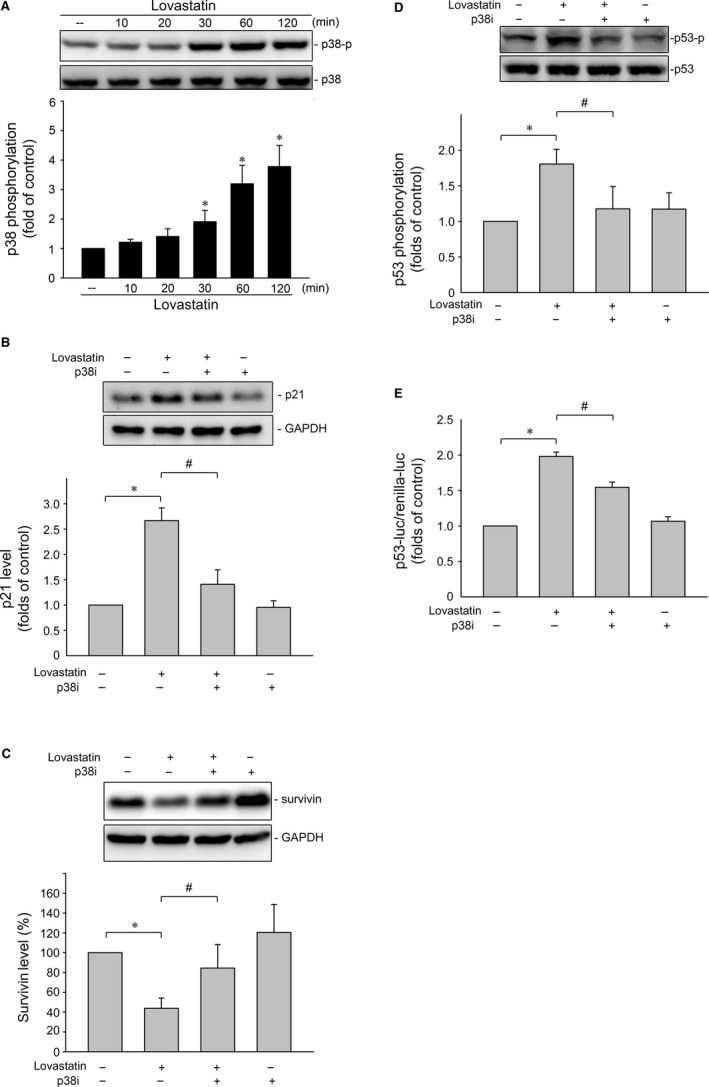
p38MAPK contributes to lovastatin‐induced p53 activation, p21 elevation and survivin reduction in MCF‐7 cells. A, Cells were treated with vehicle or lovastatin at 30 μmol/L for indicated periods. The extent of p38MAPK phosphorylation (MW 38 kD) was examined by immunoblotting. Each column represents the mean ± SEM of six independent experiments (Statistically significant differences were determined using the Kruskal‐Wallis test. *P < .05, compared with the control group). Cells were pre‐treated with p38MAPK inhibitor III (p38i) at 1 μmol/L for 30 min. After treatment, cells were stimulated with lovastatin at 30 μmol/L for another 24 h. Protein levels of p21 (B) or survivin (C) were determined by immunoblotting. Each column represents the mean ± SEM of six independent experiments (Statistically significant differences were determined using the Mann‐Whitney test. *P < .05, compared with the vehicle‐treated control group; ^#^ *P* < .05, compared with the group treated with lovastatin alone). D, Cells were pre‐treated with p38MAPK inhibitor III (p38i) at 1 μmol/L for 30 min. After treatment, cells were stimulated with lovastatin at 30 μmol/L for another 1 h. The extent of p53 phosphorylation was determined by immunoblotting. Each column represents the mean ± SEM of six independent experiments (Statistically significant differences were determined using the Mann‐Whitney test. *P < .05, compared with the vehicle‐treated control group; ^#^ *P* < .05, compared with the group treated with lovastatin alone). (E) Cells were transiently transfected with PG13‐luc (p53‐luc) plus renilla‐luc for 24 h. After transfection, cells were pre‐treated with p38MAPK inhibitor III (p38i) at 1 μmol/L for 30 min followed by the stimulant with lovastatin (30 μmol/L) for another 24 h. Reporter assay was performed as described in the ‘Materials and Methods’ section. Each column represents the mean ± SEM of five independent experiments (Statistically significant differences were determined using the Mann‐Whitney test. *P < .05, compared with the vehicle‐treated control group; ^#^ *P* < .05, compared with the group treated with lovastatin alone)

We next explored the signalling mechanisms underlying lovastatin‐induced p53 activation in MCF‐7 cells. We previously demonstrated that p38MAPK activates p53, resulting in cell death in cerebral endothelial cells,^50^ glioma cells^51^ and colorectal cancer cells.^20^ Therefore, we examined whether lovastatin affects p38MAPK phosphorylation status in MCF‐7 cells. As shown in Figure 4A, lovastatin caused a time‐dependent increase in p38MAPK phosphorylation. In contrast, p38MAPK inhibitor III (p38i), a selective ATP‐competitive p38MAPK inhibitor,^52^ significantly suppressed p21 elevation (Figure 4B) and restored survivin reduction (Figure 4C) in lovastatin‐stimulated MCF‐7 cells. Moreover, p38MAPK inhibitor III reduced lovastatin's enhancing effect in inducing p53 Ser15 phosphorylation (Figure 4D) and p53 luciferase activity (Figure 4E). These results suggest that p38MAPK signalling contributes to lovastatin's actions in MCF‐7 cells.

### 3.5 LKB1‐AMPK signalling contributes to lovastatin‐induced p38MAPK and p53 phosphorylation

**Figure 5:**
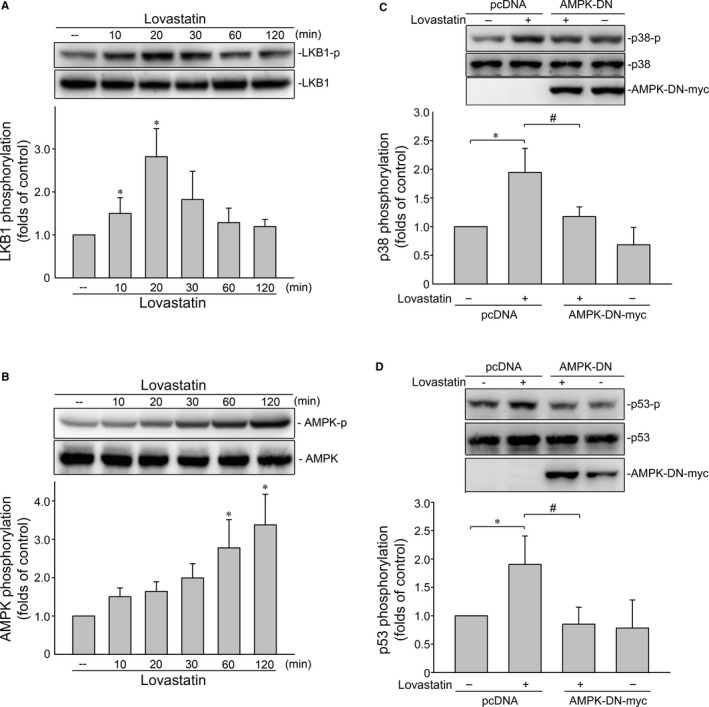
AMPK mediates lovastatin‐induced p38MAPK and p53 phosphorylation in MCF‐7 cells. Cells were treated with vehicle or lovastatin at 30 μmol/L for indicated periods. The extent of LKB1 (MW 54 kD) (A) or AMPK (MW 62 kD) (B) phosphorylation was determined by immunoblotting. Each column represents the mean ± SEM of six independent experiments (Statistically significant differences were determined using the Kruskal‐Wallis test. *P < .05, compared with the control group). Cells were transfected with pcDNA or AMPK‐DN for 48 h. After transfection, cells were treated with vehicle or lovastatin (30 μmol/L) for another 1 h. The extent of p38MAPK (C) or p53 (D) phosphorylation was determined by immunoblotting. Each column represents the mean ± SEM of four independent experiments (Statistically significant differences were determined using the Mann‐Whitney test. *P < .05, compared with the vehicle‐treated control group; ^#^ *P* < .05, compared with the group treated with lovastatin alone)

**Figure 6:**
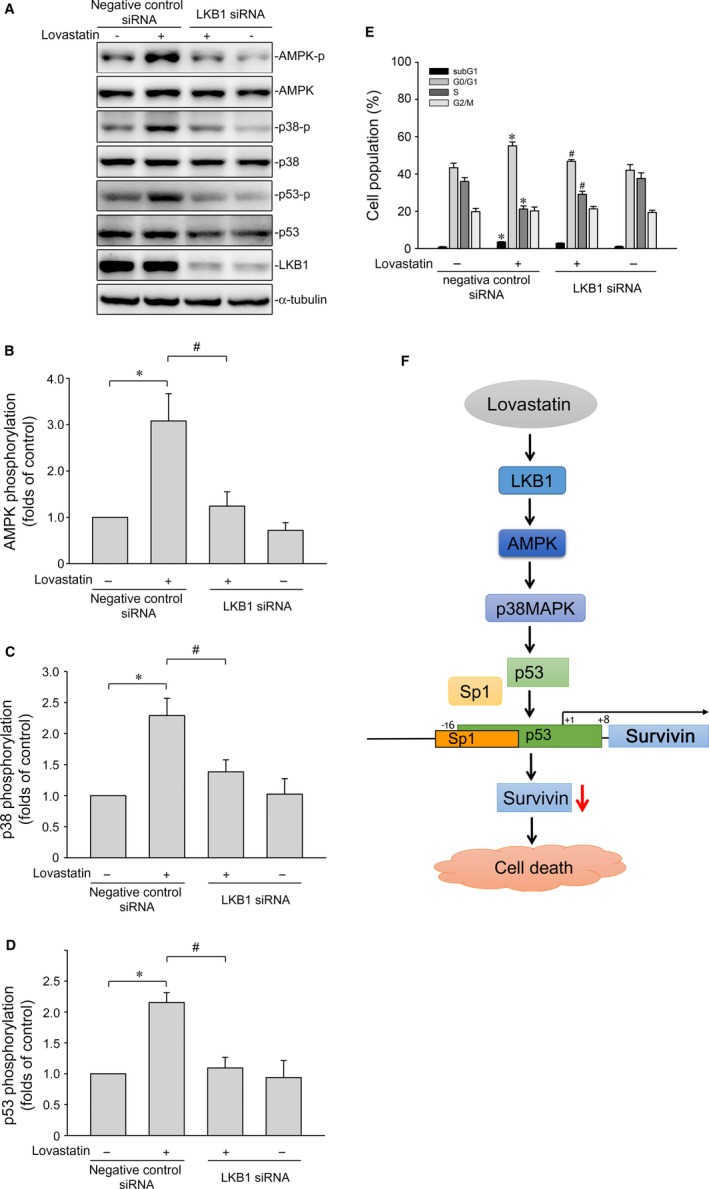
LKB1 contributes to lovastatin‐induced AMPK, p38MAPK and p53 phosphorylation in MCF‐7 cells. A, Cells were transfected with negative control siRNA or LKB1 siRNA for 48 h. After transfection, cells were treated with vehicle or lovastatin (30 μmol/L) for another 1 h. The extent of LKB1 and phosphorylation status of AMPK, p38MAPK or p53 was determined by immunoblotting. The compiled results of AMPK (B), p38MAPK (C) and p53 (D) phosphorylations are shown. Each column represents the mean ± SEM of six independent experiments (Statistically significant differences were determined using the Mann‐Whitney test. *P < .05, compared with the vehicle‐treated control group; ^#^ *P* < .05, compared with the group treated with lovastatin alone). (E) After transfection as described in (A), cells were treated with vehicle or lovastatin (30 μmol/L) for another 24 h. The percentage of propidium iodide‐stained cells in subG1, G0/G1, S and G2/M phases was analysed by flow cytometry. Each column represents the mean ± SEM of eight independent experiments (Statistically significant differences were determined using one‐way ANOVA, with Tukey's post hoc test. *P < .05, compared with the negative control siRNA‐transfected group; ^#^ *P* < .05, compared with the negative control siRNA‐transfected group in the presence of lovastatin). F, Schematic summary of the signalling pathway involved in lovastatin‐induced MCF‐7 breast cancer cell death

Growing evidence shows that serine/threonine kinase liver kinase B1 (LKB1) regulates a variety of cellular events such as cell cycle arrest, senescence and cell death.^53^ A number of different mechanisms including AMPK activation^54^ and survivin reduction^53^ mediate these effects. In addition, Hsu et al^42^ demonstrated that activation of AMPK‐p38MAPK signalling leads to survivin reduction and subsequent cell death in colorectal cancer cells. Therefore, we examined whether lovastatin's effects on MCF‐7 cells involves LKB1 or AMPK signalling. As shown in Figure 5A, lovastatin caused an increase in LKB1 phosphorylation in a time‐dependent manner. Lovastatin also time‐dependently induced AMPK phosphorylation (Figure 5B). Transfection of cells with myc‐tagged AMPK dominant negative mutant (AMPK‐DN) significantly suppressed lovastatin‐induced p38MAPK (Figure 5C) and p53 (Figure 5D) phosphorylation. We next used LKB1 siRNA strategy to establish the causal role of LKB1 in lovastatin‐induced activation of AMPK‐p38MAPK‐p53 signalling cascade. As shown in Figure 6, LKB1 knockdown by LKB1 siRNA significantly reduced AMPK (Figure 6B), p38MAPK (Figure 6C), and p53 (Figure 6D) phosphorylation in lovastatin‐stimulated MCF‐7 cells. Furthermore, LKB1 siRNA also reduced lovastatin's effects on the percentage of PI‐stained cells in the G0/G1 and S region (Figure 6E). Together these findings support the contention that lovastatin may activate the LKB1‐AMPK‐p38MAPK‐p53 signalling cascade, leading to survivin reduction and MCF‐7 cell death (Figure 6F). On the other hand, lovastatin also caused LKB1 (Figure S4) and AMPK phosphorylation (Figure S5) in mutant p53‐harbouring MDA‐MB‐231 cells. Transfection of MDA‐MB‐231 cells with LKB1 siRNA (Figure S4) or AMPK‐DN (Figure S5) significantly reduced lovastatin's inhibitory effects on survivin expression. It suggests that lovastatin‐induced survivin reduction is causally related to LKB1‐AMPK signalling in not only MCF‐7 cells, but also in MDA‐MB‐231 cells.

## 4 DISCUSSION

Statins have been used to treat hyperlipidemia and reduce cardiovascular morbidity and mortality for decades worldwide. Statins suppress cholesterol biosynthesis by targeting HMG‐CoA reductase in the hepatic mevalonate pathway. The mevalonate pathway regulates not only cholesterol production, but also a variety of cellular processes. It is likely that suppression of these pathways is responsible for statins’ pleiotropic effects, in particular, anti‐tumour properties. Statins may also exhibit anti‐tumour activities through the mechanisms independent of the cholesterol‐lowering property.^14^, ^20^, ^31^, ^32^ Accumulating epidemiologic, pre‐clinical and clinical evidence demonstrated that statins inhibit cell proliferation and cause cell apoptosis in breast cancer cells. It also reduces the risk of breast cancer recurrence.^3^, ^25^, ^55^ However, the molecular mechanisms underlying statins’ anti‐breast cancer effects remain poorly understood and to be further investigated. In this study, we demonstrated that lovastatin, a lipophilic statin, activates LKB1‐AMPK‐p38MAPK‐p53‐survivin cascade to cause cell death in MCF‐7 breast cancer cells.

Survivin reduction leads to cell cycle arrest and apoptosis in breast cancer cells.^13^ We noted in this study that lovastatin caused survivin reduction in not only MCF‐7, but also MDA‐MB‐231 and MDA‐MB‐468 cells (Figure S2). Lovastatin‐induced survivin reduction is causally related to p53 in MCF‐7 cells. It appears that p53‐mediated survivin reduction may account for lovastatin's anti‐proliferative and apoptotic effects in MCF‐7 cells. In keeping with previous studies that statins increase p21 level and suppress cancer cell proliferation,^14^, ^20^ we noted that p38MAPK mediates p21 induction in lovastatin‐stimulated MCF‐7 cells. However, lovastatin‐induced p21 up‐regulation was likely through p53‐independent pathway.^56^ Lafarga et al^57^ reported that p38MAPK induces p21 mRNA stabilization, resulting in p21 accumulation and cell cycle arrest. Inhibition of HDACs may also contribute to lovastatin's actions in elevating p21 in cancer cells.^32^ The mechanisms underlying lovastatin‐induced cyclinD1 reduction remain to be investigated. Deregulated cyclin D1 degradation appears to be responsible for the increased levels of cyclin D1 in several cancers. A number of studies^58^, ^59^ demonstrated that p38MAPK phosphorylates cyclin D1 and induces its proteasomal degradation. It raises the possibility that lovastatin activation of p38MAPK signalling not only modulates survivin and p21 as reported here, but also reduces cyclin D1 in breast cancer cells. Moreover, Zhang et al^60^ reported that knockdown cyclin D1 by siRNA strategy induces survivin reduction and cell death in cancer cells. It appears that these signalling cascades downstream of p38MAPK may converge in cell cycle arrest and cell death in breast cancer cells. Additional works are needed to characterize the interrelationship between these proteins in lovastatin‐induced breast cancer cell death.

We showed that lovastatin activates LKB1‐AMPK‐p38MAPK signalling pathway, leading to p53 phosphorylation. In addition to phosphorylation, p53 post‐translational modifications such as acetylation, ubiquitination or sumoylation also regulate its stability and transcriptional activity.^48^ Lin et al^32^ reported that statins act as HDAC inhibitors to restore the expression of silenced tumour suppressor genes in cancer cells. In addition, we previously demonstrated that HDACs inhibition leads to p53 acetylation and subsequent colorectal cancer cell death.^15^ It raises the possibility that lovastatin not only phosphorylates, but also acetylates p53 in MCF‐7 cells. Further investigations are needed to characterize the precise mechanisms of lovastatin in inducing p53 acetylation. The possibility of HDACs inhibition contributing to lovastatin‐induced p53 activation and subsequent cellular events in MCF‐7 cells is also worth to be examined.

In contrast to p53’s negative regulatory role on survivin expression, other transcription factors such as STAT3^13^ and Sp1^47^ could activate survivin promoter to up‐regulate its expression. Constitutive STAT3 phosphorylation and activation are found in numerous cancer types including breast cancer.^61^ In addition, aberrant STAT3 activation contributes to breast cancer progression.^62^ We previously demonstrated that STAT3 knockdown by STAT3 siRNA causes survivin reduction and subsequent cell death in MDA‐MB‐231 TNBC cells.^13^ Wang et al^63^ reported that simvastatin inactivates STAT3 to cause cell cycle arrest in hepatocellular carcinoma cells. MDA‐MB‐231 is a p53 mutant cell line while MCF‐7 cells retain functional p53,^64^ whereas basal STAT3 phosphorylation level is comparably higher in TNBC cells (eg, MDA‐MB‐231 or MDA‐MB‐468) as compared with luminal‐type (eg, MCF‐7) or HER2‐positive (eg, BT474) breast cancer cells.^65^ We noted that lovastatin‐reduced cell viability is accompanied by survivin reduction (Figure S2) and STAT3 dephosphorylation (Figure S3) in MDA‐MB‐231 and MDA‐MB‐468 cells. It appears that lovastatin‐induced survivin reduction also attributes to STAT3 inactivation in breast cancer cells. The underlying mechanisms of lovastatin in inactivating STAT3 in breast cancer cells remain unresolved. HDACs inhibition has been shown to activate protein tyrosine phosphatase SHP‐1, leading to STAT3 dephosphorylation in breast cancer cells.^13^ It is likely that lovastatin activates a protein tyrosine phosphatase such as SHP‐1 to dephosphorylate STAT3 and thereby cause survivin reduction in breast cancer cells. These are all worth to be further investigated.

In addition to MCF‐7 cells, we also noted that LKB1 (Figure S4) or AMPK (Figure S5) contributes to lovastatin‐induced survivin reduction in another subtype breast cancer cells, MDA‐MB‐231 cells. Vasamsetti et al^66^ reported that AMPK activation causes STAT3 inactivation. In addition, arterial injury‐induced STAT3 activation is associated with suppression of LKB1 and AMPK activity in smooth muscle cells.^67^ It raises the possibility that lovastatin activation of LKB1‐AMPK signalling cascade not only activates p53 as shown in this study, but also inactivates STAT3 in breast cancer cells. The link between LKB1‐AMPK‐p38MAPK‐p53 and AMPK‐STAT3 cascades and the differential mechanisms underlying lovastatin‐activated these two signalling pathways remain to be established. It appears that these two pathways may converge in survivin reduction, resulting in breast cancer cell death.

The mutation of p53 is the most common genetic abnormality found in nearly half of all human cancers. In contrast to wild‐type tumour‐suppressive properties, mutant p53 may gain tumour‐promoting activities, which facilitate tumour proliferation, invasion and metastasis.^68^, ^69^ It is believed that elevated mutant p53 levels in tumour cells are a consequence of increased its protein stability. Recent studies demonstrated that inhibiting mevalonate pathway by statins might destabilize and thereby degrade mutant p53, leading to tumour cell death.^68^, ^69^, ^70^, ^71^ We noted in this study that mutant p53‐harbouring MDA‐MB‐468 and MDA‐MB‐231 cells are sensitive to lovastatin. It appears that targeting mevalonate pathway may also contribute to lovastatin's apoptotic actions in breast cancer cells. These findings suggest that the mechanisms underlying lovastatin‐induced breast cancer cell death may differ among tumour subtypes or genetic background. Further investigations are needed to establish the causal role of p53 or mutant p53 in lovastatin's apoptotic actions in breast cancer cells. It will be also interesting to clarify the interplay between these signalling cascades as discussed above.

In conclusion, we demonstrated that lovastatin exhibits anti‐tumour activities via LKB1‐AMPK‐p38MAPK‐p53‐survivin cascade in luminal MCF‐7 breast cancer cells. Lovastatin may also activate LKB1‐AMPK signalling or inactivate STAT3, leading to survivin reduction and cell death in mutant p53‐harbouring MDA‐MB‐231 TNBC cells. Together these observations suggest that statin may be a potential candidate for intervention of breast cancer.

## CONFLICT OF INTEREST

None.

## AUTHOR CONTRIBUTIONS

SWH, ITC, YFH and MJH designed the experiments; SWH, CS, MCY and MJH performed the experiments; SWH, ITC, CS, MCY, YFH and MJH analysed the data; YFH and MJH wrote the paper.

## Data Availability

The data that support the findings of this study are available from the corresponding author upon reasonable request.
