# Supplementary material for: Lovastatin‐mediated MCF‐7 cancer cell death involves LKB1‐AMPK‐p38MAPK‐p53‐survivin signalling cascade
Source: J Cell Mol Med. 2019 Dec 10;24(2):1822–36. doi: 10.1111/jcmm.14879 (PMC6991643; doi:10.1111/jcmm.14879)
Supplement: Supplementary file 1 [file JCMM-24-1822-s001.pdf]

Supplement Information

Supplement Figures

Supplement Fig. S1

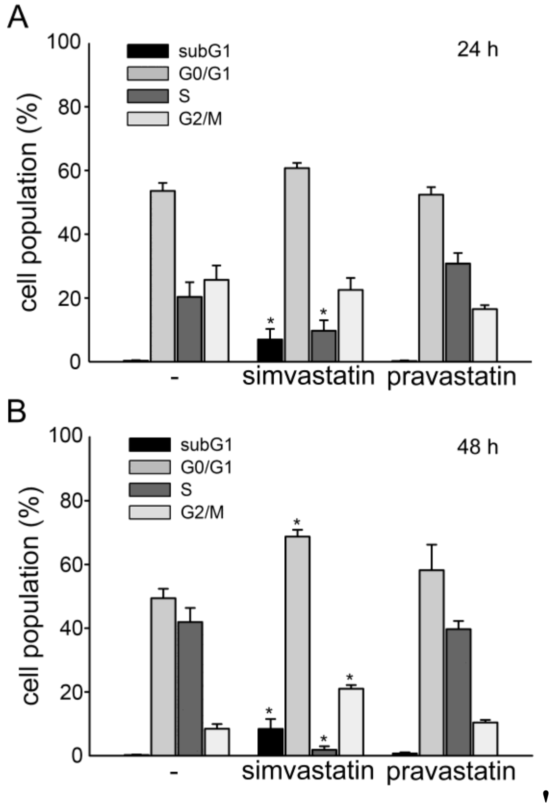

Supplement Fig. S2

A

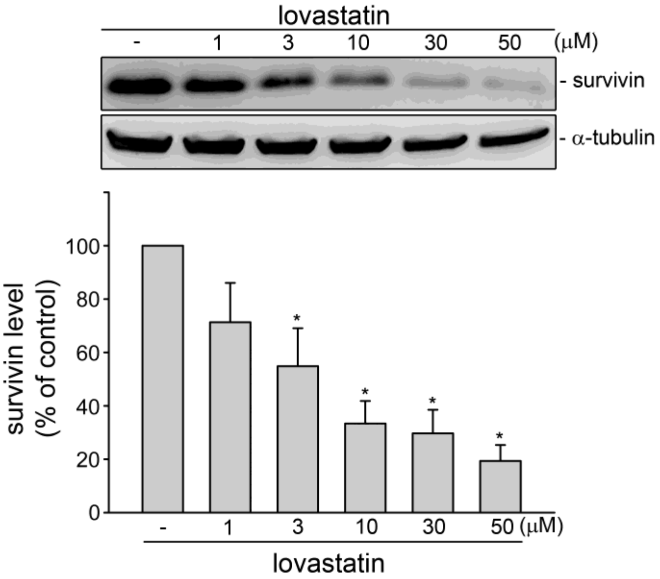

B

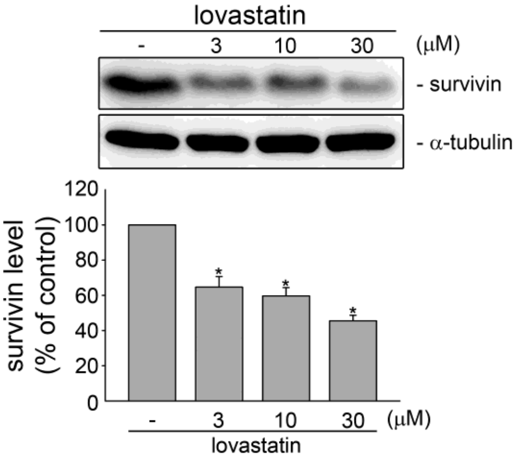

Supplement Fig. S3

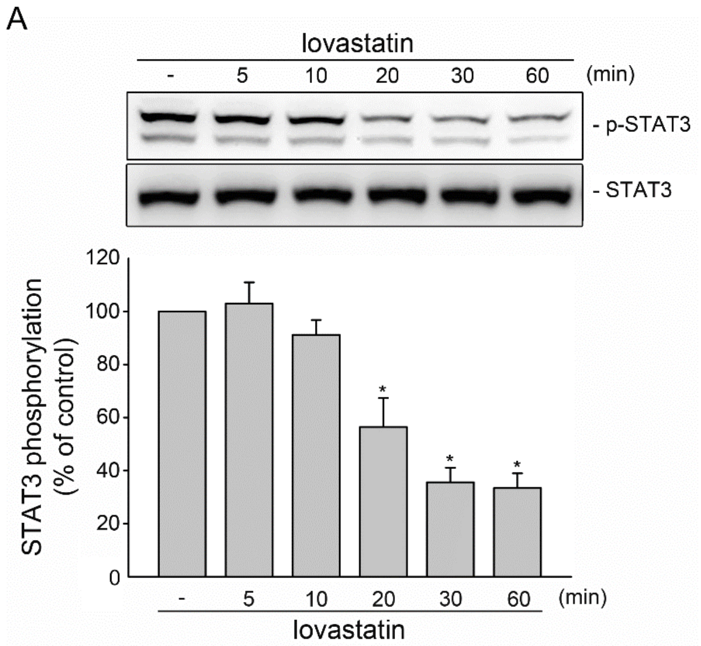

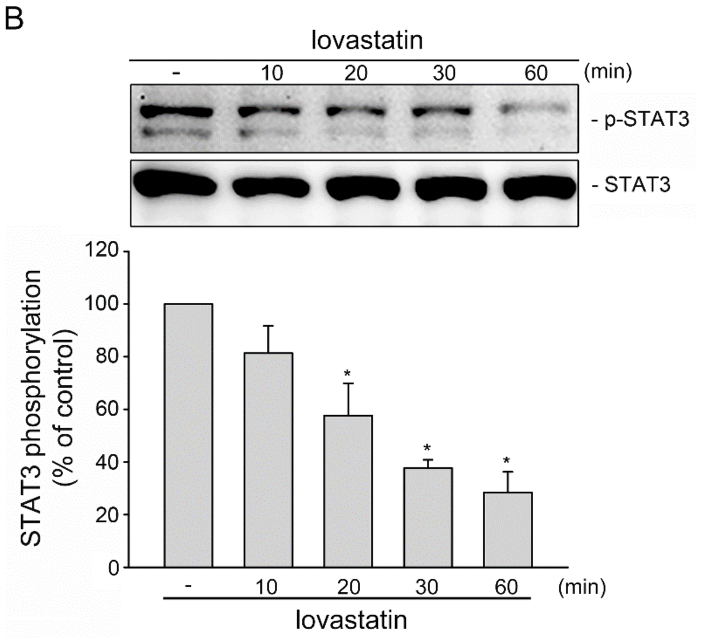

Supplement Fig. S4

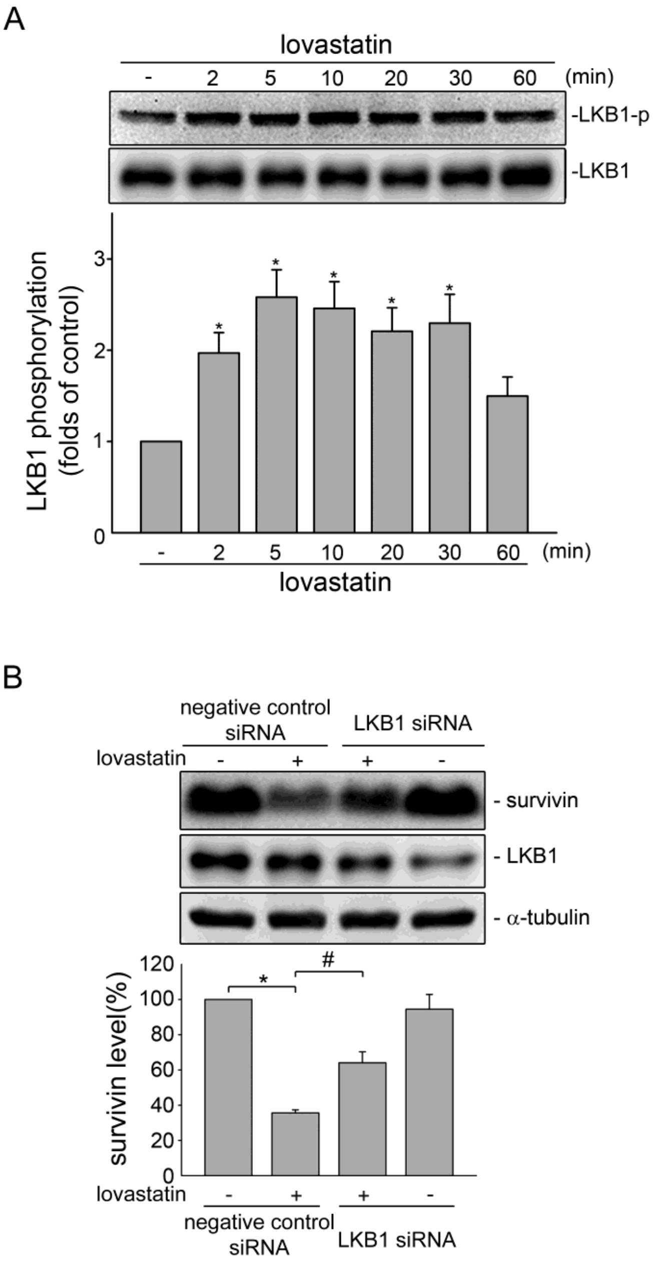

Supplement Fig. S5

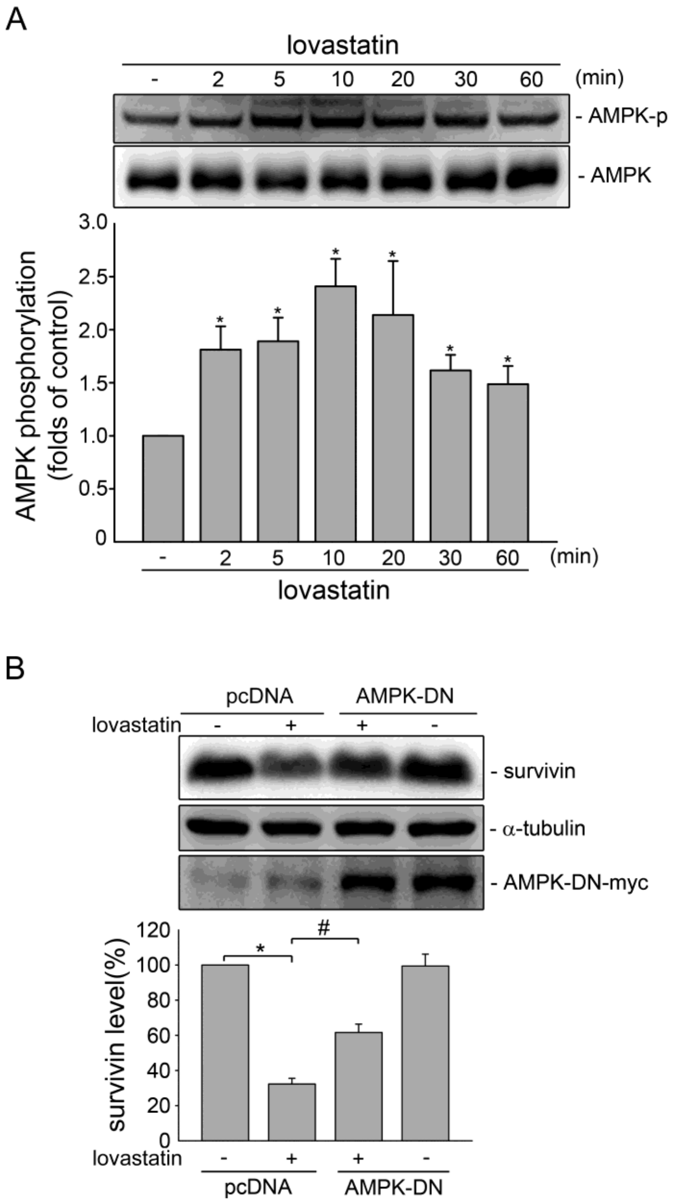

Supplement Figure legends

Supplement Figure S1. Effects of simvastatin and pravastatin on cell cycle progression or apoptosis in MCF-7 cells. MCF-7 cells were treated with vehicle, simvastatin (30 μM) or pravastatin (30 μM) for 24 h (A) or 48 h (B). The percentage of propidium iodide-stained cells in subG1, G0/G1, S, and G2/M phases was analyzed by flowcytometry as described in the "*Materials and methods*" section. Each column represents the mean ± S.E.M. of four independent experiments (Statistically significant differences were determined using one-way ANOVA, with Tukey's post-hoc test. *$p <$ 0.05, compared with the control group).

Supplement Figure S2. Lovastatin caused survivin reduction in MDA-MB-468 and MDA-MB-231 cells.

MDA-MB-468 (A) and MDA-MB-231 (B) cells were treated with vehicle or lovastatin at indicated concentrations for 24 h. Protein levels of survivin (MW 16 kDa) and α-tubulin (MW 52 kDa) were determined by immunoblotting. Each column represents the mean ± S.E.M. of six independent experiments (Statistically significant differences were determined using the Kruskal-Wallis test. *$p < 0.05$, compared with the control group).

Supplement Figure S3. Lovastatin reduced STAT3 phosphorylation in MDA-MB-468 and MDA-MB-231 cells. MDA-MB-231 (A) and MDA-MB-468 (B) cells were treated with vehicle or lovastatin at 30 μM for the indicated periods. The phosphorylation status of STAT3 Tyr705 (MW 79, 86 kDa) was determined by immunoblotting. Each column represents the mean ± SEM of five independent experiments. Statistically significant differences were determined using the Kruskal–Wallis test. *P < 0.05, compared with the control group.

Supplement Figure S4. LKB1 contributes to lovastatin-induced survivin reduction in MDA-MB-231 cells. (A) Cells were treated with vehicle or lovastatin at 30 μM for indicated periods. The extent of LKB1 phosphorylation was determined by immunoblotting. Each column represents the mean ± S.E.M. of six independent experiments (Statistically significant differences were determined using the Kruskal-Wallis test. *$p < 0.05$, compared with the control group). (B) Cells were transfected with negative control siRNA or LKB1 siRNA for 48 h. After transfection, cells were treated with vehicle or lovastatin (30 μM) for another 24 h. The extent of survivin was determined by immunoblotting. Each column represents the mean ± S.E.M. of six independent experiments (Statistically significant differences were determined using the Mann-Whitney test. *$p < 0.05$, compared with the vehivle-treated control group; #*p*

< 0.05, compared with the group treated with lovastatin alone).

Supplement Figure S5. AMPK contributes to lovastatin-induced survivin reduction in MDA-MB-231 cells. (A) Cells were treated with vehicle or lovastatin at 30 μM for indicated periods. The extent of AMPK phosphorylation was determined by immunoblotting. Each column represents the mean ± S.E.M. of six independent experiments (Statistically significant differences were determined using the Kruskal-Wallis test. *$p < 0.05$, compared with the control group). (B) Cells were transfected with pcDNA or AMPK-DN for 48 h. After transfection, cells were treated with vehicle or lovastatin (30 μM) for another 24 h. The extent of survivin was determined by immunoblotting. Each column represents the mean ± S.E.M. of six independent experiments (Statistically significant differences were determined using the Mann-Whitney test. *$p < 0.05$, compared with the vehivle-treated control group; #$p < 0.05$, compared with the group treated with lovastatin alone).

Service Report

Customer Information:

Institute	Graduate Institute of Medical Sciences,<br>Taipei Medical University
Name	Dr. Hsu, Ming-Jen
Report Date	2019.08.15

Service Description:

**Service Name**	Mycoplasma Detection
**Service Summary**	PCR based mycoplasma detection. Mycoplasma contamination is indicated by the presence of bands at approximately 370 bp -500 bp.
**Brand of Detection Kit**	Abm
**Catalog Number of Detection Kit**	G238
**Name of Detection Kit**	PCR Mycoplasma Detection Kit
**Lot Number of Detection Kit**	0194844636001
**Sample(s) Information**	A254 - A258

Detection Result:

Service item	Method	Result
Mycoplasma Detection	PCR	No Mycoplasma contamination

PCR 電泳結果圖

M: 100 bp DNA Ladder

N: Negative control

P: Positive control

A235: 549

A236: 231

A237: 468

A238: MCF7

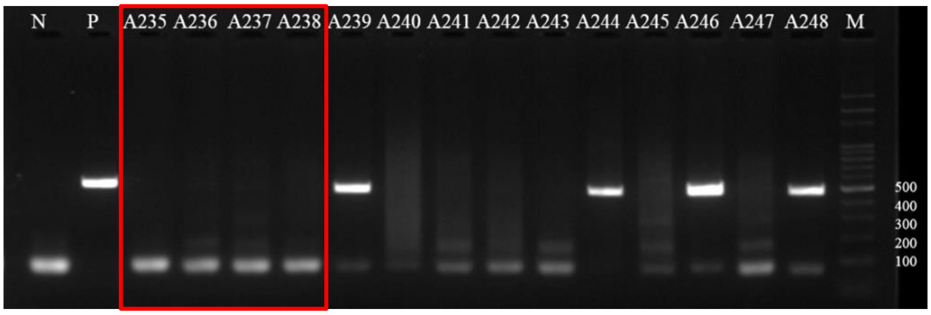

Suggestions: We recommend to use **InvivoGen, Plasmocin™(Catalog number: ANT-MPP)** to prevent Mycoplasma contamination.

InvivoGen, Plasmocin™ product information: https://www.invivogen.com/plasmocin

Mycoplasma Screening assay

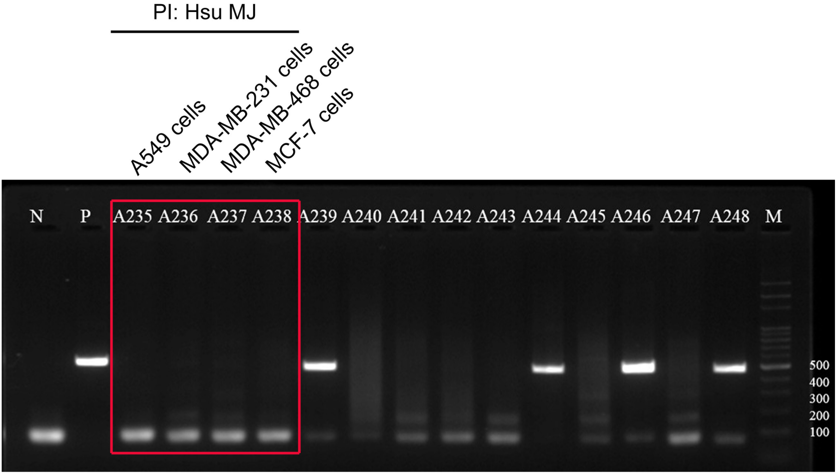

M: 100bp DNA ladder marker ; N: negative control; P:positivi control

Genelabs Life Science Corp.
12F.-6, No.3, Yuanqu St.,
Nangang Dist.,
Taipei City 115, Taiwan
TEL: +886-2-26557678
FAX: +886-2-26557572
E-mail: cellid@genelabs.com.tw

Cell Line DNA Typing Report

Case Number: CID20190140

Report Date: 07/09/2019

Sample Information:

i. Applicant Name: 許銘仁 Hsu Ming-Jen

ii. Institution: 臺北醫學大學藥理學科 Department of Pharmacology, Taipei Medical University

iii. Sample Description: MCF-7

iv. Sample type: Cell Pellet

v. Sample Received Date: 06/26/2019

Test Description:

DNA of the sample is extracted by Roche MagNA Pure Compact System.

DNA conc. = 151.9 ng/μl; OD260/280 = 2.03 ; OD260/230 = 2.14

The STR loci are amplified by Promega GenePrint® 24 System.

The CE analysis is performed on ABI PRISM 3730 GENETIC ANALYZER.

The raw data is analyzed by GeneMapper® Software V3.7.

The STR analysis is operated and reported by Mission Biotech.

This report is issued by:

Liang Kuei Chang — Laboratory Director

James Chung — General Manager

STR Analysis Result:

ANSI/ATCC ASN-0002 STR Loci	Repeat Numbers	Extended STR Loci	Repeat Numbers	Extended STR Loci	Repeat Numbers
**D5S818**	11,12	D3S1358	16,16	DYS391*	N/A
**D13S317**	11,11	D1S1656	15.3,15.3	D8S1179	10,14
**D7S820**	8,9	D2S441	10,14	D12S391	18,20
**D16S539**	11,12	D10S1248	14,14	D19S433	13,14
**vWA**	14,15	Penta E	7,12	FGA	23,25
**TH01**	6,6	D18S51	14,14	D22S1045	15,16
**Amelogenin**	X	D2S1338	23,23	Case Number: CID20190140<br>Test Date: 07/05/2019	
**TPOX**	12,12	Penta D	12,12		
**CSF1PO**	10,10	D21S11	30,30		

Allele Report

Case Number: CID20190140

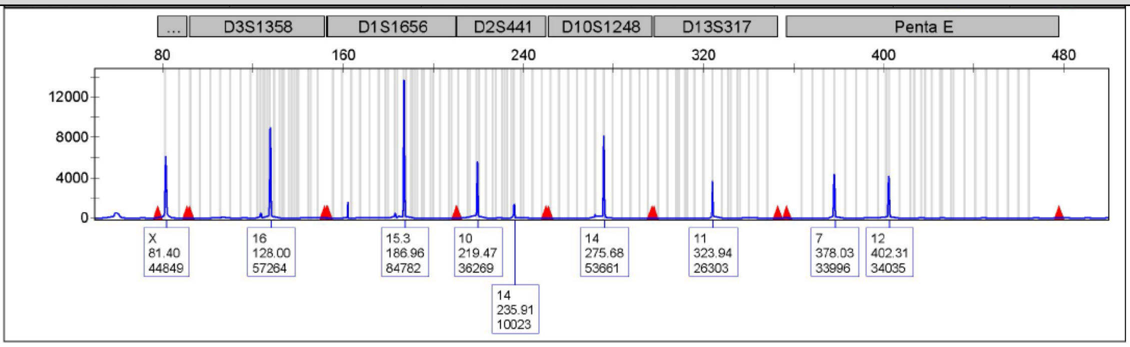

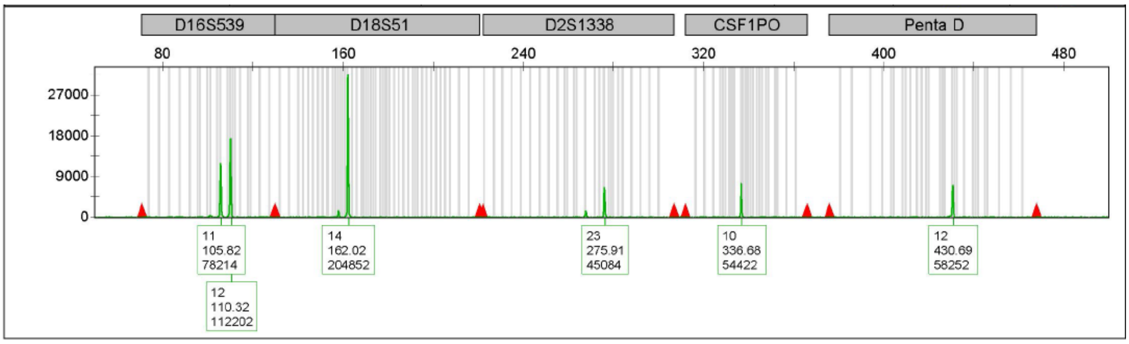

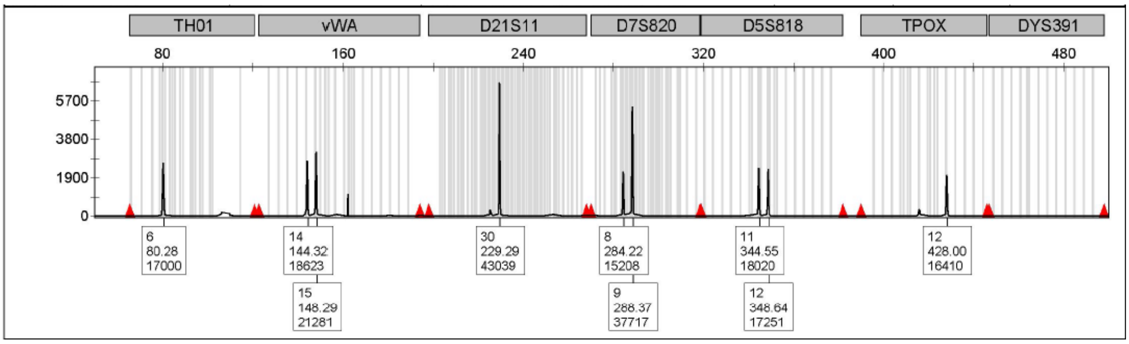

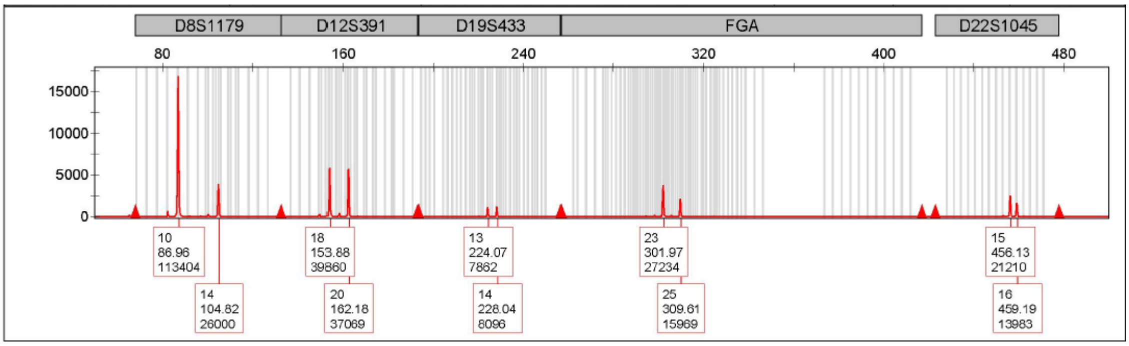

	Dye	Sample File Name	Marker	Allele	Size	Height	Area
1	B,1	008_A02_MCF-7.fsa	AMEL	X	81.4	6228	44849
2	B,2	008_A02_MCF-7.fsa	D3S1358	16	128	8999	57264
3	B,3	008_A02_MCF-7.fsa	D1S1656	15.3	186.96	13750	84782
4	B,4	008_A02_MCF-7.fsa	D2S441	10	219.47	5648	36269
5	B,5	008_A02_MCF-7.fsa	D2S441	14	235.91	1535	10023
6	B,6	008_A02_MCF-7.fsa	D10S1248	14	275.68	8209	53661
7	B,7	008_A02_MCF-7.fsa	D13S317	11	323.94	3749	26303
8	B,8	008_A02_MCF-7.fsa	Penta E	7	378.03	4419	33996
9	B,9	008_A02_MCF-7.fsa	Penta E	12	402.31	4257	34035
10	G,1	008_A02_MCF-7.fsa	D16S539	11	105.82	12108	78214
11	G,2	008_A02_MCF-7.fsa	D16S539	12	110.32	17464	112202
12	G,3	008_A02_MCF-7.fsa	D18S51	14	162.02	31483	204852
13	G,4	008_A02_MCF-7.fsa	D2S1338	23	275.91	6684	45084
14	G,5	008_A02_MCF-7.fsa	CSF1PO	10	336.68	7466	54422
15	G,6	008_A02_MCF-7.fsa	Penta D	12	430.69	7217	58252
16	Y,1	008_A02_MCF-7.fsa	TH01	6	80.28	2683	17000
17	Y,2	008_A02_MCF-7.fsa	vWA	14	144.32	2738	18623
18	Y,3	008_A02_MCF-7.fsa	vWA	15	148.29	3216	21281
19	Y,4	008_A02_MCF-7.fsa	D21S11	30	229.29	6629	43039
20	Y,5	008_A02_MCF-7.fsa	D7S820	8	284.22	2226	15208
21	Y,6	008_A02_MCF-7.fsa	D7S820	9	288.37	5457	37717
22	Y,7	008_A02_MCF-7.fsa	D5S818	11	344.55	2409	18020
23	Y,8	008_A02_MCF-7.fsa	D5S818	12	348.64	2337	17251
24	Y,9	008_A02_MCF-7.fsa	TPOX	12	428	2038	16410
25	R,1	008_A02_MCF-7.fsa	D8S1179	10	86.96	16947	113404
26	R,2	008_A02_MCF-7.fsa	D8S1179	14	104.82	4019	26000
27	R,3	008_A02_MCF-7.fsa	D12S391	18	153.88	5947	39860
28	R,4	008_A02_MCF-7.fsa	D12S391	20	162.18	5849	37069
29	R,5	008_A02_MCF-7.fsa	D19S433	13	224.07	1198	7862
30	R,6	008_A02_MCF-7.fsa	D19S433	14	228.04	1230	8096
31	R,7	008_A02_MCF-7.fsa	FGA	23	301.97	3802	27234
32	R,8	008_A02_MCF-7.fsa	FGA	25	309.61	2243	15969
33	R,9	008_A02_MCF-7.fsa	D22S1045	15	456.13	2552	21210
34	R,10	008_A02_MCF-7.fsa	D22S1045	16	459.19	1702	13983

The comparison between case result and STR profile database:

Cell Name	Locus name / STR Repeat Number									Match (%)	STR Profile Database*
	D5S818	D13S317	D7S820	D16S539	VWA	TH01	AM	TPOX	CSF1PO		
Test Sample	11,12	11,11	8,9	11,12	14,15	6,6	X,X	12,12	10,10		
MCF-7	11,12	11,11	8,9	11,12	14,15	6,6	X,X	9,12	10,10	94	DSMZ
MCF7	11,12	11,11	8,9	11,12	14,15	6,6	X,X	9,12	10,10	94	DSMZ
KPL-1	11,12	10,11	8,9	11,12	14,15	6,6	X,X	9,12	10,10	89	DSMZ

* Online STR Analysis is performed on https://www.atcc.org/STR_Database.aspx (ATCC) or https://www.dsmz.de/services/services-human-and-animal-cell-lines/online-str-analysis.html (DSMZ)

* This table just shows the top 10 cell lines within database those matches are ≥80%, and if there isn't any match ≥80%, it just shows the cell line which scores highest match with the test sample. If necessary, please utilize the online STR analysis on ATCC/DSMZ website for detailed comparison.

The interpretation of comparison:

Depending on the comparison, the highest 94% match is between the test sample and two cell lines (MCF-7, MCF7) within database, and there is another cell line those match is ≥80%.

Referring to ASN-0002-2011 (Authentication of Human Cell Lines: Standardization of STR Profiling), Cell line samples matching at ≥80% of alleles across the eight (8) core loci are said to be related, allowing authentication of the sample being tested. STR profiles are said to be unrelated when they match at <55% of alleles. STR profiles matching at 55-80% of alleles may be related and warrant further investigation as there may be overlap with a small number of cell lines that show marked genetic instability and slip below the 80% threshold.

- ***This STR analysis service is for research purposes only, and any medical usage is not allowed.***

Genelabs Life science Corporation

www.genelabs.com.tw

Phone:(02)26557678, (04)22633813, (06)2094380

Freecall:0800-231914, 0800-094380

REPORT DOWNLOAD

Cell Line DNA Typing Report

Mission Biotech
10F-3, No.3, Yuanchi Street
Nangang, Taipei
Taiwan 115
Tel: 886 2 26557128
Email: service@missionbio.com.tw

Case Number: CID20130040
Report Date: 05/03/2013

Sample Information：

i. Applicant Name: Department of Pharmacology, School of Medicine, College of Medicine, Taipei Medical University
ii. Institution: Ming-Jen Hsu
iii. Sample Description: MDA-MB231
iv. Sample type: Cell pellet
v. Sample Received Date: 04/23/2013

Allele table for the tested cell DNA

STR Locus	Repeat Numbers
D5S818	12
D13S317	13
D7S820	8,9
D16S539	12
vWA	15,18
TH01	7,9.3
Amelogenin	X
TPOX	8,9
CSF1PO	12,13
D21S11	30,33.2
Mouse	-

Test Description：

Case Number: CID20130040
Test Date: 05/03/2013

Sample was extracted by Roche MagNA Pure Compact System.
DNA conc.= 124.2 ng/ul
OD260/280 = 2.03
OD260/230 = 2.15

This test was performed by using the Promega StemElite™ ID System and analyzed by ABI PRISM 3730 GENETIC ANALYZER and GeneMapper® Software V3.7.

Verified by:
Laboratory Director(Title)

Liang Kuei Chang

05/06/2013

Allele Report

Case Number: CID20130040

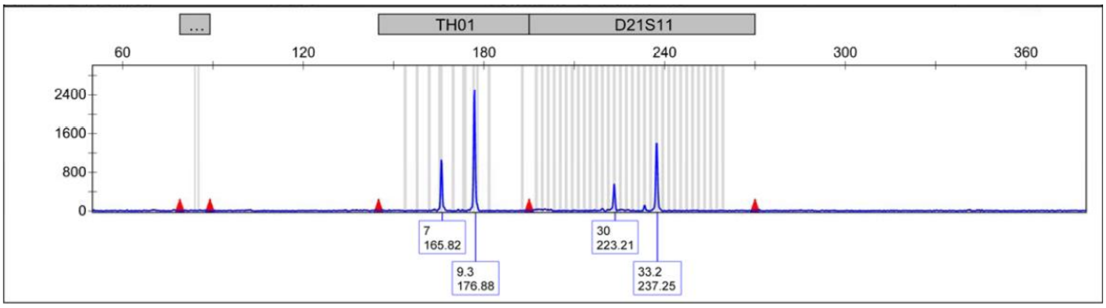

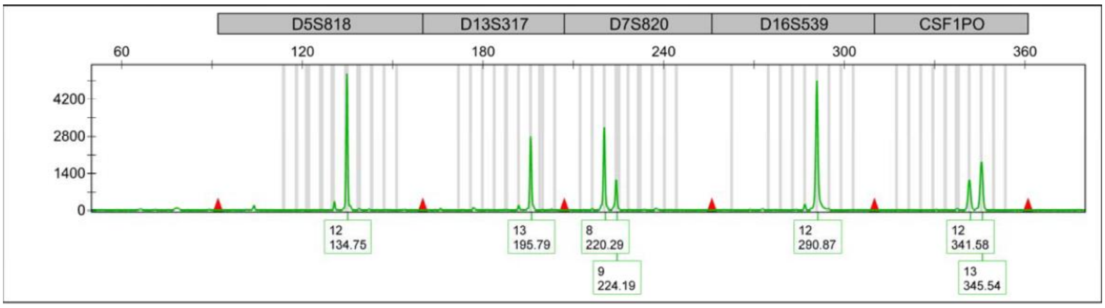

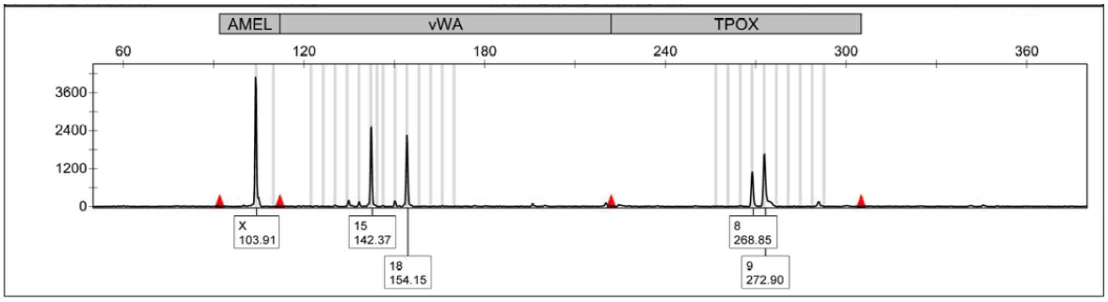

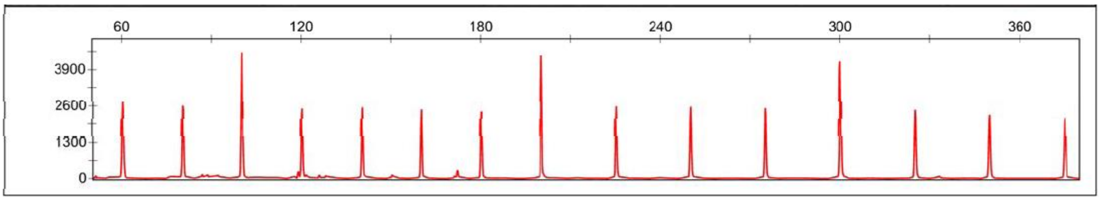

	Dye	Sample File Name	Marker	Allele	Size	Height	Area
1	B,1	047_B12_ MDAMB231.fsa	TH01	7	165.82	1058	4552
2	B,2	047_B12_ MDAMB231.fsa	TH01	9.3	176.88	2485	10489
3	B,3	047_B12_ MDAMB231.fsa	D21S11	30	223.21	564	2477
4	B,4	047_B12_ MDAMB231.fsa	D21S11	33.2	237.25	1398	6844
5	G,1	047_B12_ MDAMB231.fsa	D5S818	12	134.75	5177	19429
6	G,2	047_B12_ MDAMB231.fsa	D13S317	13	195.79	2783	11789
7	G,3	047_B12_ MDAMB231.fsa	D7S820	8	220.29	3153	14684
8	G,4	047_B12_ MDAMB231.fsa	D7S820	9	224.19	1165	6149
9	G,5	047_B12_ MDAMB231.fsa	D16S539	12	290.87	4901	28753
10	G,6	047_B12_ MDAMB231.fsa	CSF1PO	12	341.58	1162	7735
11	G,7	047_B12_ MDAMB231.fsa	CSF1PO	13	345.54	1842	12601
12	Y,1	047_B12_ MDAMB231.fsa	AMEL	X	103.91	4100	15268
13	Y,2	047_B12_ MDAMB231.fsa	vWA	15	142.37	2521	9916
14	Y,3	047_B12_ MDAMB231.fsa	vWA	18	154.15	2269	9040
15	Y,4	047_B12_ MDAMB231.fsa	TPOX	8	268.85	1108	5612
16	Y,5	047_B12_ MDAMB231.fsa	TPOX	9	272.9	1669	9860

Allelic Ladder

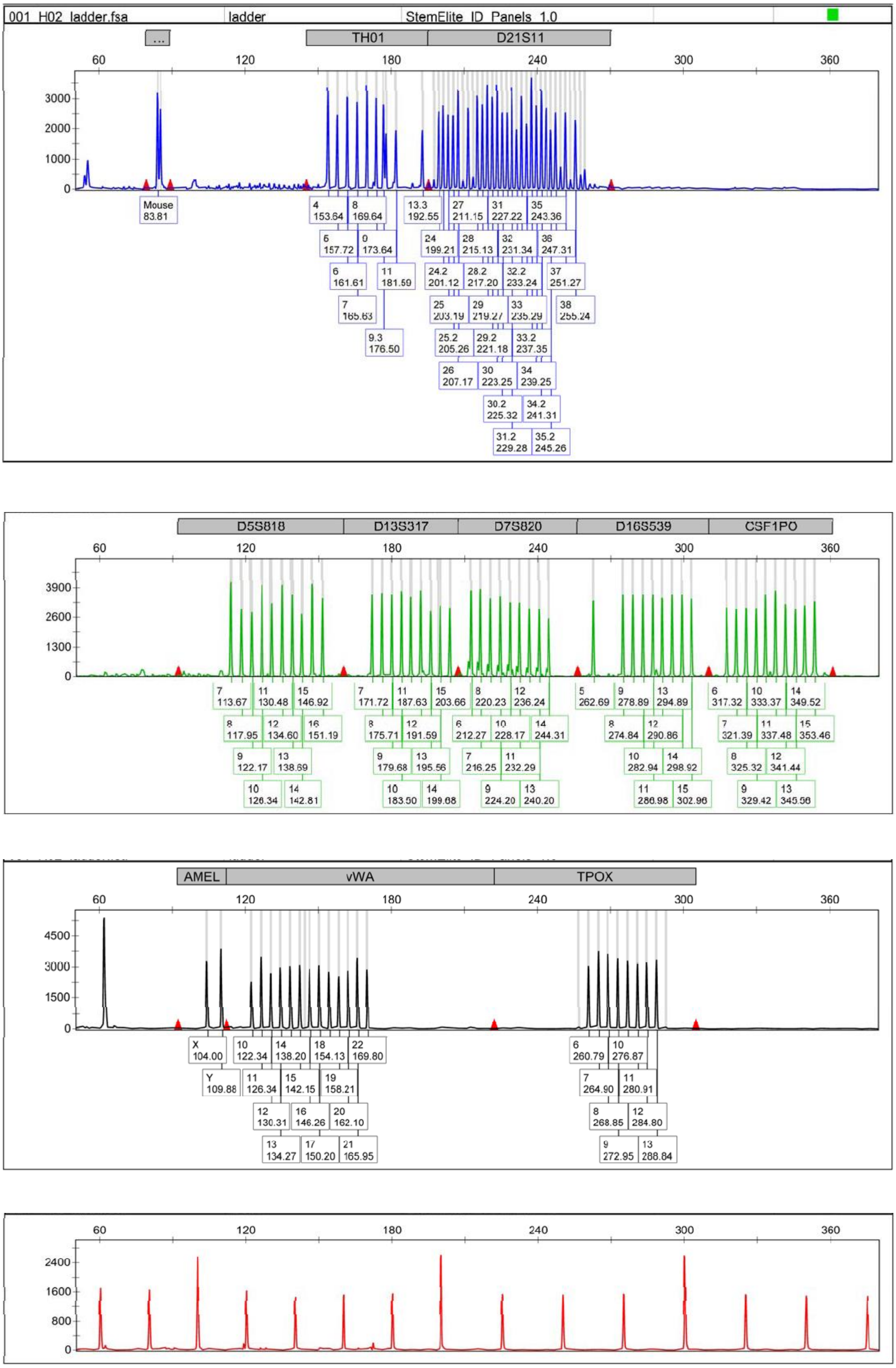

- *This STR analysis testing service is for research purposes only, and is not to be used for clinical diagnosis or applications involving humans.*
- *This test is just for typing the specific loci of the applied sample. The applicants may have to compare the results with the database of some bioresource institutes such as ATCC, JCRB or DSMZ by themselves.*

Genelabs Life science www.genelabs.com.tw
Phone:(02)26557678, (04)22633813, (06)2094380
Freecall:0800-231914,0800-094380
